# COVID-19 Surveillance Updates in US Metropolitan Areas: Dynamic Panel Data Modeling

**DOI:** 10.2196/28737

**Published:** 2022-02-24

**Authors:** Theresa B Oehmke, Charles B Moss, James F Oehmke

**Affiliations:** 1 Department of Civil and Environmental Engineering University of California, Berkeley Berkeley, CA United States; 2 Institute of Food and Agricultural Sciences University of Florida Gainesville, FL United States; 3 Department of Emergency Medicine Feinberg School of Medicine Northwestern University Chicago, IL United States

**Keywords:** surveillance system, COVID-19, coronavirus, Sars-CoV-2, Houston, dynamic panel data model, speed, jerk, acceleration, 7-Day persistence, modeling, data, surveillance, monitoring, public health, United States, transmission, response

## Abstract

**Background:**

Despite the availability of vaccines, the US incidence of new COVID-19 cases per day nearly doubled from the beginning of July to the end of August 2021, fueled largely by the rapid spread of the Delta variant. While the “Delta wave” appears to have peaked nationally, some states and municipalities continue to see elevated numbers of new cases. Vigilant surveillance including at a metropolitan level can help identify any reignition and validate continued and strong public health policy responses in problem localities.

**Objective:**

This surveillance report aimed to provide up-to-date information for the 25 largest US metropolitan areas about the rapidity of descent in the number of new cases following the Delta wave peak, as well as any potential reignition of the pandemic associated with declining vaccine effectiveness over time, new variants, or other factors.

**Methods:**

COVID-19 pandemic dynamics for the 25 largest US metropolitan areas were analyzed through September 19, 2021, using novel metrics of speed, acceleration, jerk, and 7-day persistence, calculated from the observed data on the cumulative number of cases as reported by USAFacts. Statistical analysis was conducted using dynamic panel data models estimated with the Arellano-Bond regression techniques. The results are presented in tabular and graphic forms for visual interpretation.

**Results:**

On average, speed in the 25 largest US metropolitan areas declined from 34 new cases per day per 100,000 population, during the week ending August 15, 2021, to 29 new cases per day per 100,000 population, during the week ending September 19, 2021. This average masks important differences across metropolitan areas. For example, Miami’s speed decreased from 105 for the week ending August 15, 2021, to 40 for the week ending September 19, 2021. Los Angeles, San Francisco, Riverside, and San Diego had decreasing speed over the sample period and ended with single-digit speeds for the week ending September 19, 2021. However, Boston, Washington DC, Detroit, Minneapolis, Denver, and Charlotte all had their highest speed of the sample during the week ending September 19, 2021. These cities, as well as Houston and Baltimore, had positive acceleration for the week ending September 19, 2021.

**Conclusions:**

There is great variation in epidemiological curves across US metropolitan areas, including increasing numbers of new cases in 8 of the largest 25 metropolitan areas for the week ending September 19, 2021. These trends, including the possibility of waning vaccine effectiveness and the emergence of resistant variants, strongly indicate the need for continued surveillance and perhaps a return to more restrictive public health guidelines for some areas.

## Introduction

Almost a year and a half after the first case of the disease caused by the novel coronavirus SARS-CoV2 was recorded in the United States, the country has reported over 42 million cases, over 680,000 deaths, 88 million initial unemployment claims filed from March 21, 2020, to the present, and an estimated 30,000 additional deaths attributable to pandemic-related unemployment [1,2]. Additionally, up to 30% of COVID-19 survivors and some asymptomatic individuals may be subject to sequalae including a deterioration in health-related quality of life that may last 9 months or longer [3-6]. Despite the unprecedented pandemic, the US government has not implemented national COVID-19 restrictions, instead leaving it to states and metropolitan areas to determine what public health measures are appropriate in their context, based on unclear guidelines [7-12]. This has led to a patchwork of measures and sometimes diverging national, state, and local guidance [7-9]. Additionally, unclear and divergent public health recommendations, among other factors, have likely affected public acceptance of measures such as social distancing, face masking, and crowd avoidance [13-15]. For example, despite the evidence that masks help prevent the spread of the virus, on March 6, protestors held a mask-burning rally on the steps of the Idaho state capitol [16], the first conviction in a plot to kidnap Michigan Governor Whitmer for imposing a mask mandate was handed down in August of 2021 [17], and Florida Governor DeSantis’s ban of local mask mandates has been subject to court challenges and likely appeals [18].

After enduring shutdowns for most of the 2020-21 winter, most US metropolitan areas began reopening in early- to mid-March, 2021 [16,19,20], despite contrary guidance and concerns of explosive growth after reopening [21,22]. Reopening is at least chronologically associated with public desensitization to COVID-19 news and lack of compliance with health guidance [23]. Since June 2021, the emergence of the delta variant has led to a global third wave of COVID-19 cases including in the Unites States, with 98.4% of new cases in the Unites States comprising lineage B.1.617.2 [24]. There is significant concern that reopening has led to an upsurge and possibly explosive growth in the pandemic, especially as new variants spread across the country, and if either health policy or social acceptance of policy reduces or eliminates social distancing. The ramifications of the Delta and other variants for the reemergence of explosive growth under reopening conditions and with limited vaccination are unclear [25]. Thus, it is imperative to have timely and objective surveillance at local levels to inform public health decisions, including metrics such as the 7-day persistence rate that inform questions around where on the epidemiological curve the pandemic currently lies—whether it is in the state of explosive growth, peaking and declining, or that it is peaking with a plateau [26,27]. A large 7-day persistence value is indicative of explosive growth.

This report provides surveillance data relevant to the question of whether the pandemic is exploding, peaking, or plateauing for the 25 largest US metropolitan areas. Specifically, it provides weekly data for the 6 weeks ending on September 19, 2021, on the novel metrics of speed, acceleration, and jerk, and the 7-day persistence effect. This captures information relevant to the determination of a possible peak and reversal in the most recent Delta-fueled wave of COVID-19 in the United States.

## Methods

We calculated the speed, acceleration, jerk, and 7-day persistence rate by applying dynamic panel data modeling and additional methods introduced by [7,26]. These methods have been previously applied globally at the country level [26-31] and in the United States at the state level [28] and comprise the basis for the Global SARS-CoV-2 Surveillance Project [32]. This paper updates the findings by Oehmke et al [27], who previously applied these methods to US metropolitan areas.

The metrics used in the paper are speed, acceleration, jerk, and persistence. Speed is the number of new metropolitan area cases per day per 100,000 population and is a measure of how fast the pandemic is growing from day to day. To address differences in reporting across different days of the week, we calculate speed (and other indicators) on a weekly basis for 7-day ISO (International Organization for Standardization) weeks [33]. Acceleration is the week-over-week change in weekly average speed and is the primary indication of whether the pandemic is getting worse (positive acceleration) or better (negative acceleration or deceleration) from week to week. Jerk is the change in acceleration. A positive jerk signifies increasing acceleration—not only is the pandemic getting worse, but it is getting worse more rapidly this week than last. A large positive jerk can be associated with super-spreading events, emergence of a new variant, policy shift, or other change that affects the underlying infection rates. A negative jerk indicates a declining acceleration possibly including a shift from positive to negative acceleration, and if associated with a policy shift, may indicate policy success. The 7-day persistence rate is the number of cases per 100,000 today that are statistically attributable to cases 7 days ago. A positive 7-day persistence rate can signify the presence of linked super-spreader events, the emergence of a new variant, or continued policy ineffectiveness. During the first year of the pandemic, persistence was associated with mega-spreader events (eg, when on a weekend people were infected at a sports event, religious gathering, political rally, etc, they go to another event the next weekend and infect more people, who then go to another event, leading to a persistent “echo forward” effect of the original infection [27]). Mathematically, persistence case numbers that are close to or exceeding 100% of total cases lead to rapid or explosive growth in the number of cases. A small positive persistence may indicate a diminishing pandemic. A negative number indicates that the high number of cases last week is associated with a lower number of cases this week, suggesting that the high number of cases was an aberration rather than an indication of a persistent issue in the metropolis. A negative 7-day persistence rate is indicative of a slowing pandemic, whether via a natural progression or through public health control measures.

We collected data from USAFacts for the week of 2021 ending August 1, 2021, through the week ending September 19, 2021. Using the US Census Bureau definitions of metropolitan area, we determined the various counties comprising the 25 largest US metropolitan areas and collected data on the total (cumulative) number of COVID-19 cases in each county for each day. We preprocessed the data only by creating a variable containing the number of new cases per county per day to show the change in the cumulative case count from the prior day to that day (first difference). We used data from the first 2 weeks, ie, the weeks ending August 1, 2021, and August 8, 2021, to create lagged values used in the analysis, resulting in a sample period of 6 weeks from the week ending August 15, 2021, to the week ending September 19, 2021, for which we had a complete data set. We analyzed the data using STATA/MP, version 17.0 (Stata Corp LLC); the dynamic panel data modeling is accomplished using STATA’s “xtabond” procedure. We report the weekly average speed, acceleration, jerk, and 7-day persistence effect for the most recent 6 weeks of 2021. The results for the prior weeks of 2021 are available from the authors upon request.

We also report the vaccination rates in California and Florida, which are useful in interpreting the results. Vaccination data are taken from the USAFacts website. Multimedia Appendix 1 contains additional methodological detail.

## Results

Table 1 reports the population-weighted average results for the 25 metropolitan areas. Speed increased from 34.03 cases per day per 100,000 persons during the week ending August 15, 2021, to a peak of 36.84 during the week ending September 5, 2021, before declining to 29.01 for the week ending September 19, 2021. Acceleration started at a positive 3.96, then declined and turned negative during the weeks ending September 12, 2021, and September 19, 2021. Jerk was negative for the first 5 weeks of the period, before turning positive during the week ending September 19, 2021. The positive jerk coupled with a negative acceleration (deceleration) means that the deceleration is slowing, which is potentially indicative of a slow decline or upcoming plateau in the number of cases. Persistence started at 1.67 new cases per day per 100,000 that were statistically attributable to cases from the prior week, during the week ending August 15, 2021. Persistence increased to a peak of 2.22 the week ending August 29, 2021, before declining to a value of 1.06 the week ending September 19, 2021.

**Table 1:** Population-weighted averages of reported COVID-19 incidents for the 25 most populous metropolitan areas in the United States.

	Population-weighted averages			
Week ending	Speed (reported cases per day per 100,000 people)	Acceleration (reported cases per day per day per 100,000 people)	Jerk (reported cases per day per day per day per 100,000 people)	Persistence (number of cases during 7-day period due to cases in pervious 7-day period)
August 15, 2021	34.03	3.96	-2.89	1.67
August 20, 2021	36.27	2.24	-1.72	1.91
August 29, 2021	36.80	.53	-1.71	2.22
September 5, 2021	36.84	.04	-.49	2.17
September 12, 2021	31.89	-4.95	-4.99	1.26
September 19, 2021	29.01	-2.88	2.07	1.06

Disaggregating the results by metropolitan area reveals a variety of patterns over the 6 weeks. We present an illustrative selection of these patterns graphically; complete results in tabular form are available in Multimedia Appendix 2 [34-38].

**Figure 1:**
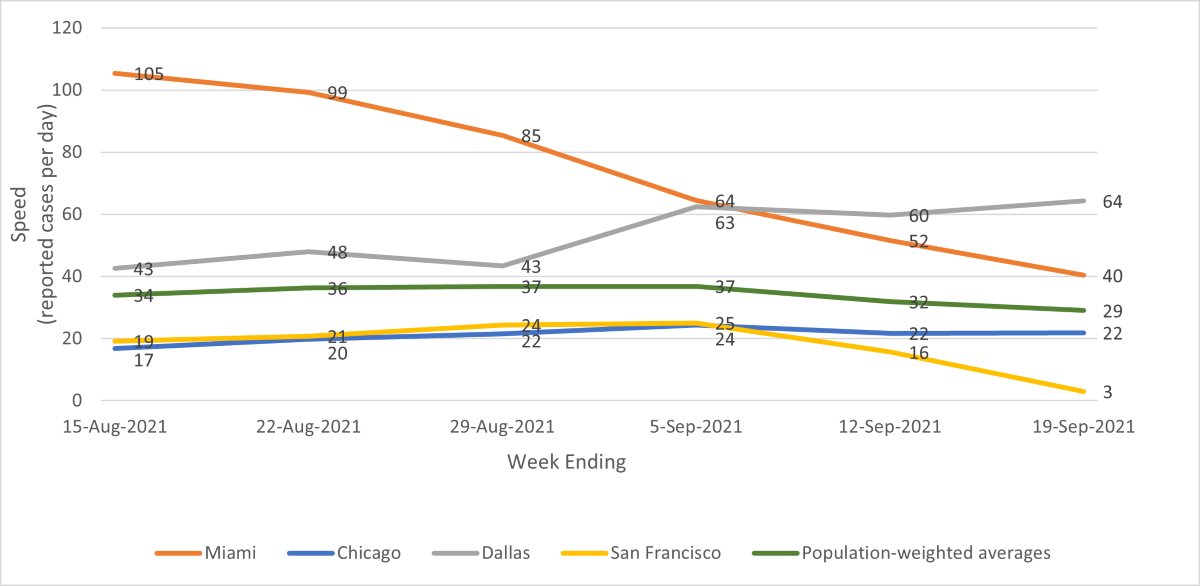
Illustrative patterns for speed; metro average, Miami, Chicago, Dallas, and San Francisco; weeks ending August 15, 2021, to September 19, 2021.

Figure 1 shows illustrative temporal patterns for speed. The population-weighted average showed a slight increase in speed through the week ending September 5, 2021, then a slight decrease. Chicago followed the national pattern of a slight increase over the first 3 or 4 weeks of the sample period, and then a slight decrease in speed. Other cities following this pattern include New York, Houston, Atlanta, Philadelphia, Phoenix, Boston, San Francisco, Riverside, Orlando, Tampa, Seattle, San Diego, and Portland. Miami started off the sample period at its peak with a high rate of speed, which declined significantly over the sample period especially in the last 2 weeks; Tampa and Orlando followed the national pattern of peaking in mid-August but had significant decreases in speed over the last 2 weeks, similarly to Miami. Los Angeles also saw a significant decline in speed over the period, but from a lower starting point. San Francisco and San Diego, while mirroring the national peak, declined rapidly in the end of the sample period to significantly decrease their speed, with San Francisco and San Diego reaching single-digit speeds. San Antonio experienced a small decrease in speed over the sample period. Dallas, Washington DC, Detroit, Minneapolis, Baltimore, Charlotte, St. Louis, and Portland saw slightly increasing speed over the period.

**Figure 2:**
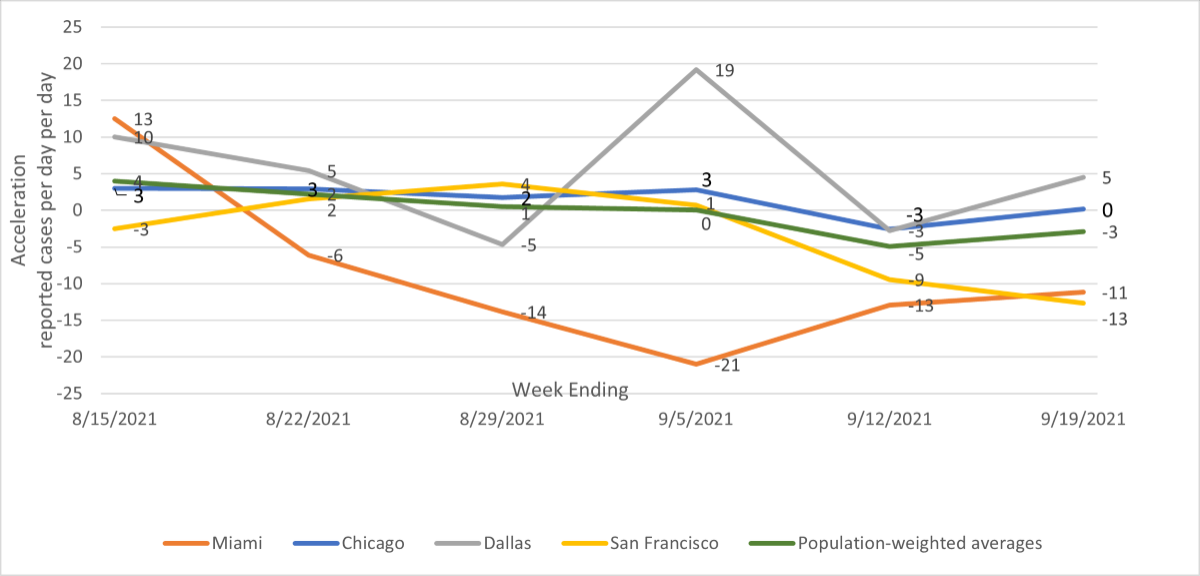
Illustrative patterns for acceleration; metro average, Miami, Chicago, Dallas, and San Francisco; weeks ending August 15, 2021, to September 19, 2021.

Figure 2 shows illustrative temporal patterns for acceleration. The population-weighted average acceleration started slightly positive and ended slightly negative. Chicago’s acceleration mirrored this pattern before ending at essentially zero for the week ending September 19, 2021. Other cities following this pattern include New York, Houston, Atlanta, Philadelphia, Phoenix, Boston, San Francisco, Riverside, Orlando, Tampa, Seattle, San Diego, and Portland. Miami started with positive acceleration, but then began a rapid deceleration (negative acceleration) that is reflected in a still declining number of cases. Los Angeles, San Francisco, Orlando, Tampa, San Diego, Atlanta, and Riverside also ended the sample period with large decelerations, although the deceleration in San Francisco and the other California areas occurred primarily in the last 2 weeks of the sample period. Dallas, Washington DC, Detroit, Minneapolis, Baltimore, St. Louis, Charlotte, and Portland had some amount of fluctuation in acceleration, but ended the period with small positive accelerations.

**Figure 3:**
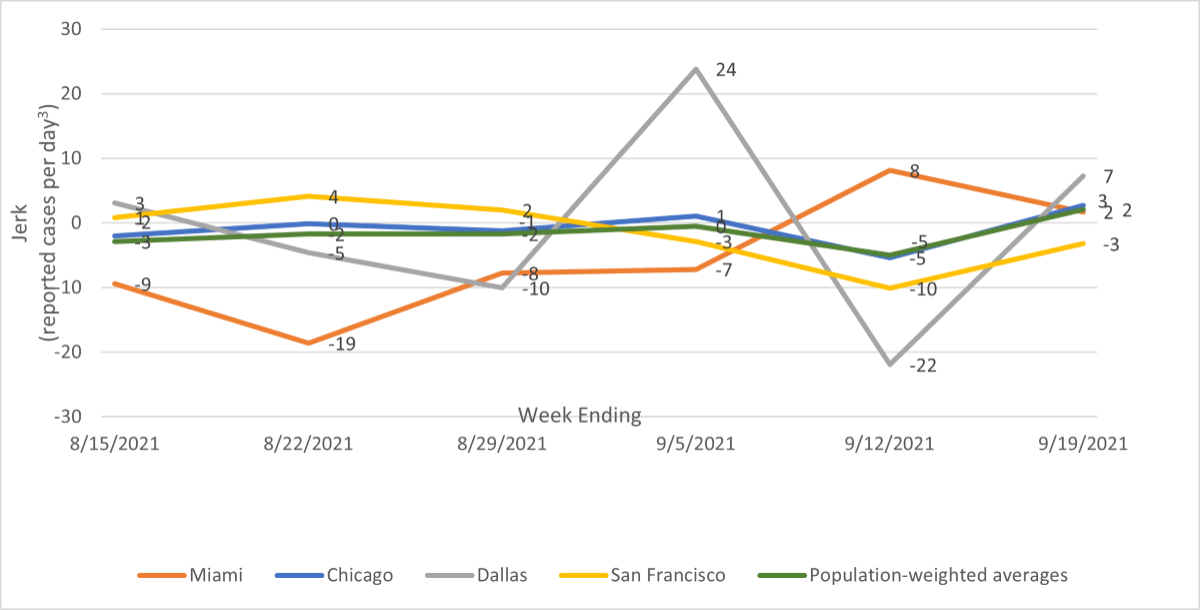
Illustrative patterns for jerk; metro average, Miami, Chicago, Dallas, and San Francisco; weeks ending August 15, 2021, to September 19, 2021.

Figure 3 shows illustrative temporal patterns for jerk—the change in acceleration. The population-weighted average jerk changes little over the sample period: it is very slightly negative the week ending August 15, 2021, and very slightly positive the week ending September 19, 2021. Since acceleration was negative (deceleration) that week, the positive jerk means that the speed is still decreasing but less rapidly. In other words, the latest wave of the pandemic has peaked, but at a national level, it is not decreasing rapidly. Chicago follows almost exactly the population-weighted average. New York, Boston, Detroit, Seattle, Minneapolis, Denver, and Baltimore were mostly similar. St. Louis inverted the average pattern, starting with small negative jerk, moving into a positive jerk, but ending the sample period with a small negative jerk. Dallas, Houston, Washington DC., Philadelphia, Phoenix, Charlotte, San Antonio, and Portland showed large fluctuations in jerk. Miami had negative jerk for the weeks ending August 15, 2021, through September 5, 2021, but jerk turned positive for the weeks ending September 12, 2021, and September 19, 2021. Los Angeles, Atlanta, San Francisco, Riverside, San Diego, Tampa, and Orlando moved from positive or near-zero jerk to large negative jerk.

**Figure 4:**
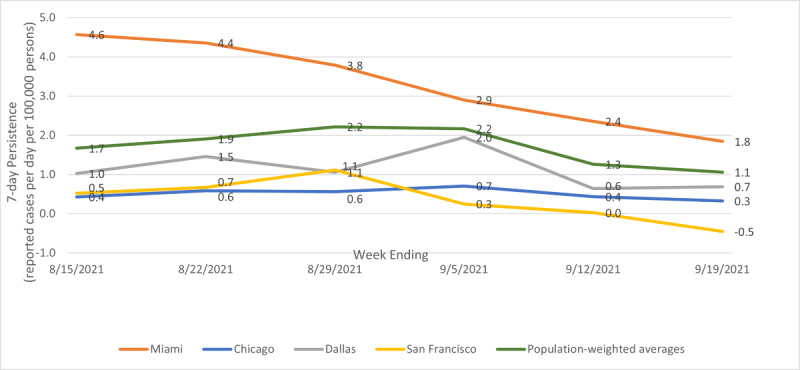
Illustrative patterns for persistence; metro average, Miami, Chicago, Dallas, and San Francisco; weeks ending August 15, 2021, to September 19, 2021.

Figure 4 shows illustrative temporal patterns for persistence, the number of daily new cases statistically attributable to new cases 7 days previously.

The metropolitan average persistence started out at 1.7 new cases per day per 100,000 statistically attributable to new cases 7 days prior, peaked at 2.2 the week ending August 29, 2021, and then declined at 1.1 the week ending September 19, 2021. As a percentage of speed, metro average persistence peaked at 6.0% the week ending August 29, 2021, and then declined to 3.7% the week ending September 19, 2021. Of the 25 largest metropolitan areas, 22 (88%) followed a similar pattern.

Exceptions to the pattern were Washington DC, Charlotte, and St. Louis. Charlotte had a definite peak the week ending September 5, 2021, and a decline the following 2 weeks, ending the sample period with a slightly higher persistence than at the start. Washington DC was similar to Charlotte but with lower numbers and a muted peak. St. Louis had a relatively high and flat persistence rate. As a percentage of total new cases, St. Louis had values from 20% to 26% over the sample period, which was notably higher than most other cities.

Table 2 provides comparisons across the 25 largest US metropolitan areas for the week ending September 19, 2021. We report the surveillance metrics of speed, acceleration, jerk, and persistence for each metropolitan area, ranked by speed. Charlotte, NC has the fastest speed at 73 new cases per day per 100,000 population, positive acceleration and jerk, and the second-highest persistence at 6.94 new cases the week ending September 19, 2021 (meaning that 6.94 new cases per day are statistically attributable to new cases in the prior week). Tampa, Orlando, and Miami have negative acceleration, consistent with their falling numbers of new cases over the entire sample period. St. Louis has the highest persistence at 7.01, meaning that over 21% of new cases during the week ending September 12, 2021, were statistically attributable to new cases the prior week. This is nearly 6 times the population-weighted average rate of 3.6%. At the bottom of the table, Riverside, San Diego, Los Angeles, and San Francisco all had single-digit speed and negative acceleration, jerk, and persistence.

The correlation coefficient between the prior week’s persistence number and the current week’s speed is 0.577 (*P*<.001).

**Figure 5:**
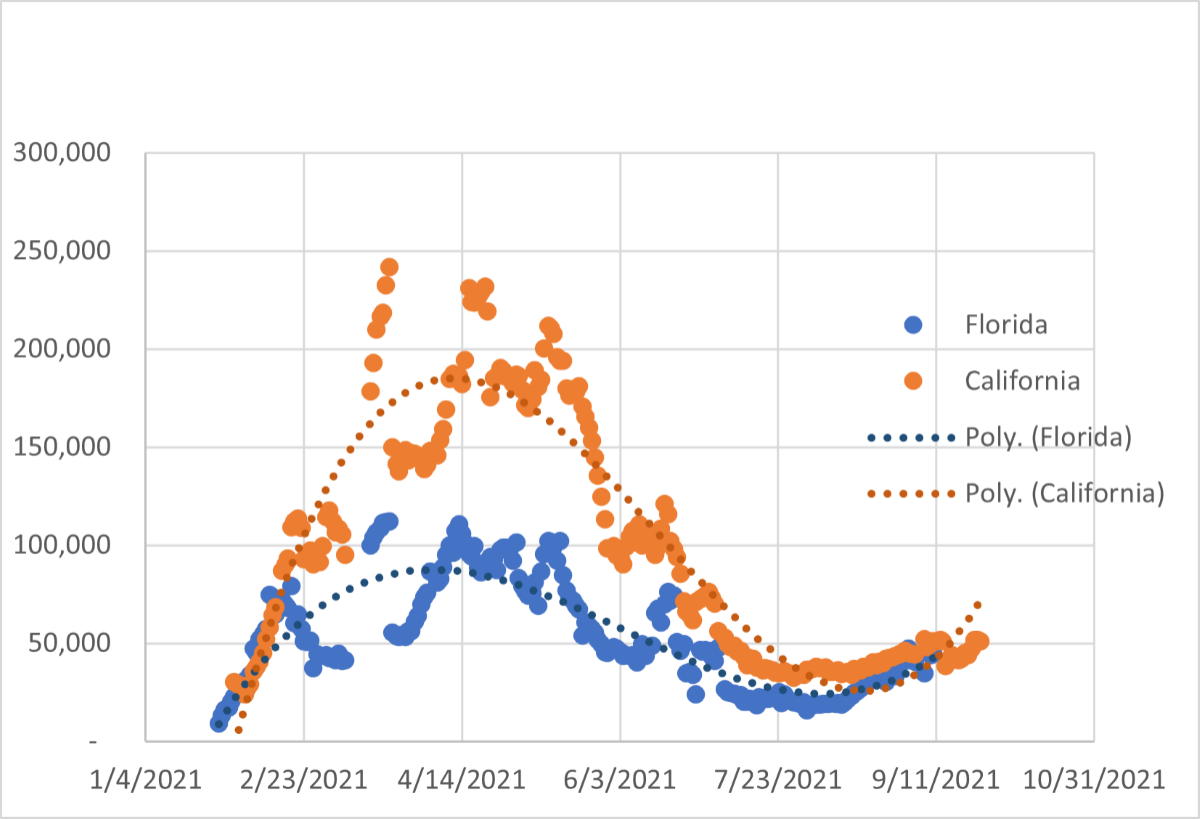
Change in fully vaccinated populations, Florida and California; 7-day moving averages and fitted cubic polynomial. Data sourced from USAFacts. Poly: polynomial.

For additional context in interpreting these results, we present vaccination rate data from California and Florida in Figure 5. Both California and Florida saw the number of new vaccinations per day peak in mid-April and decline through the end of July. Both states had a smaller “second wave” of vaccinations that began about mid-August.

**Table 2:** Speed, acceleration, jerk, and 7-Day persistence for the 25 most populous metropolitan areas in the United States, September 13-19, 2021.

Metropolitan area	Population (number of people in each metropolitan area)	Speed (reported cases per day per 100,000 people)	Acceleration (reported cases per day per day per 100,000 people)	Jerk (reported cases per day per 100,000 people)	Persistence (number of cases during 7-day period due to cases in pervious 7-day period)
Charlotte	2,636,883	73	11	22	6.94
Dallas	7,573,136	64	5	7	0.69
San Antonio	2,550,960	61	1	2	0.07
Tampa	3,194,831	56	-19	-2	1.10
Houston	7,066,141	56	6	24	0.66
Orlando	2,608,147	49	-12	7	5.18
Miami	6,166,488	40	-11	2	1.84
Phoenix	4,948,203	40	1	8	0.14
Atlanta	6,020,364	36	-8	-2	1.86
St. Louis	2,803,228	33	0	-3	7.01
Minneapolis	3,640,043	32	7	8	2.01
Portland	2,492,412	31	2	8	0.13
Seattle	3,979,845	31	0	3	0.27
Denver	2,967,239	28	1	1	1.06
Washington DC	5,371,160	24	5	6	3.39
Philadelphia	5,378,441	23	2	4	0.68
Boston	4,873,019	23	4	5	0.83
Detroit	4,319,629	23	2	2	0.80
Chicago	9,458,539	22	0	3	0.33
New York City	19,216,182	21	-1	1	0.70
Baltimore	2,800,053	16	1	2	1.11
Riverside	4,650,631	7	-20	-16	-0.41
San Diego	3,338,330	6	-17	-9	-0.25
Los Angeles	13,214,799	4	-13	-10	-0.07
San Francisco	4,731,803	3	-13	-3	-0.45

## Discussion

### Principal Findings

The 25 largest metropolitan areas are exhibiting 4 or 5 distinct patterns of COVID-19. This is not surprising as areas differ in vaccination rates, health guidelines, especially around mask mandates, and public compliance with both mandates and voluntary guidelines. Overall, the third wave of COVID-19 in the largest US metropolitan areas, associated with the Delta variant, seems to have peaked. However, how and even whether the number of new cases declines will vary by metropolitan area.

The majority of the metropolitan areas are exhibiting indications that the Delta wave peaked in mid-August, and current indications are positive. However, declines in new cases since the peak have been slow, and the epidemiological curve seems to be flattening more than rapidly declining.

Charlotte, NC exhibited the highest speed among the metropolitan areas. This high speed is consistent with low vaccination rates: less than half of North Carolina residents are fully vaccinated, and there are 272 outbreaks in nursing homes [39,40]. The next 6 areas—Dallas, San Antonio, Tampa, Houston, Orlando, Miami—are in Texas and Florida, 2 states with legislation prohibiting mask mandates [39,40].

Miami, Tampa, and Orlando are exceptions and have exhibited the biggest declines in new cases per day per 100,000 population. However, they started from very high numbers of new cases, so the current pandemic speed is significantly above average. California’s metropolitan areas also experienced significant decelerations in September, although not as large as Florida’s. However, California started at lower levels than did Florida; after deceleration, Los Angeles, San Francisco, Riverside, and San Diego all had single-digit speed during the week ending September 19, 2021. These metropolitan areas are likely close to defeating COVID-19.

The Florida pattern—the highest initial speeds during the week ending August 15, 2021, followed by the largest decline in speeds throughout the sample period for Miami and during the weeks ending September 12 and September 9, 2021, for Orlando and Tampa—is atypical among metropolitan areas. Anecdotally, the rapid decline is associated with an increase in vaccinations as the public responded to the increasing speed [41]. Figure 5 shows the daily change in the number of fully vaccinated persons in Florida as a 7-day moving average and a cubic polynomial curve. The daily increase (7-day moving average) in the number of fully vaccinated persons in Florida bottomed on August 12, 2021, and then began increasing, which is chronologically consistent with the declines in speed later in August and September. California’s metropolitan areas also experienced significant decelerations in September, although not as large as Florida’s. California also experienced an upswing in the rate of vaccination starting in mid-August, although proportionately the upswing was smaller than in Florida. Hence there is prima facie evidence that is consistent with the vaccination hypothesis. However, further research is necessary to draw any firm conclusions.

Throughout the analysis, St. Louis has presented uniquely, exhibiting a very mild trough instead of a peak, and a high persistence. We found a high and statistically significant correlation between last week’s persistence and this week’s speed; in the case of St. Louis, the high persistence number may indicate that the metropolitan area remains at risk of the pandemic worsening in the next few weeks.

However, examination of the novel surveillance metric of persistence revealed that no metropolitan area showed indications of explosive growth. The relatively small persistence numbers for the majority of metropolitan areas further suggest that the infection pattern for the Delta variant, compared with that of the Alpha or Beta variants, is less reliant on mega-spreader events and more likely to be spread by an infected person coming into casual contact with a susceptible person, or perhaps by encountering multiple susceptible persons sequentially rather than at a single event. This is heuristically consistent with the findings that Delta has a longer infectious period and higher viral shedding loads than earlier variants [42].

### Conclusions

The novel surveillance metrics in this paper help paint a more complete picture of the COVID-19 pandemic in the largest US metropolitan areas. Acceleration and jerk provide additional information as to the direction of the pandemic (ie, where a metropolitan area lies on the epidemiological curve). Perhaps most importantly, last week’s 7-day persistence is highly and significantly correlated with this week’s speed, which suggests that 7-day persistence may add significant predictive power to the existing set of surveillance metrics. This also indicates the need for further research to clarify and refine the predictive power of the 7-day persistence metric.

In mid-August, the third or Delta wave of COVID-19 peaked in most, but not all, of the 25 largest US metropolitan areas. Washington DC and St. Louis are exceptions to this peak. Following the peak, new cases declined significantly in metropolitan areas in California and Florida, although from high starting values in Florida. In California, the Los Angeles, San Francisco, Riverside, and San Diego metropolitan areas have single-digit speed (new cases per day per 100,000), which, at least for the moment, establishes control over the pandemic. However, most metropolitan areas are showing only small decreases in the number of new cases after the peak. This leads to the conclusion that more needs to be done in these areas to control the pandemic, and additional monitoring, surveillance, and improved prediction are necessary.

## Appendix

Multimedia Appendix 1 Weekly surveillance summary 2021 for 25 largest U.S. metropolitan areas.

Multimedia Appendix 2 Extended Methodology Section.

## Abbreviations

ISO: International Organization for Standardization
