# Supplementary material for: COVID-19 Surveillance Updates in US Metropolitan Areas: Dynamic Panel Data Modeling
Source: JMIR Public Health Surveill. 2022 Feb 24;8(2):e28737. doi: 10.2196/28737 (PMC8914733; doi:10.2196/28737)
Supplement: Multimedia Appendix 2 [file publichealth_v8i2e28737_app2.docx]

Annex--Extended Methodology Section

We calculated the speed, acceleration, jerk, and 7-day persistence rate by applying dynamic panel data modeling and additional methods introduced by [7,26]. These methods have been previously applied globally at the country level [26-30], in the U.S. at the state level [28], and comprise the basis for the Global SARS-Cov-2 Surveillance Project [32]. This paper updates Oehmke et al, who previously applied these methods to U.S. metropolitan areas [27].

Speed is the number of new metropolitan area cases per day per 100,000 population and is a measure of how fast the pandemic is growing from day to day. To address differences in reporting across different days of the week we calculate speed (and other indicators) on a weekly basis for 7-day ISO weeks [33]. Acceleration is the week-over-week change in weekly average speed and is the primary indication of whether the pandemic is getting worse (positive acceleration) or better (negative acceleration or deceleration) from week to week. Jerk is the change in acceleration. A positive jerk signifies increasing acceleration—not only is the pandemic getting worse, but it is getting worse more rapidly this week than last. A large positive jerk can be associated with a super-spreading event(s), emergence of a new variant, policy shift, or other change that affects the underlying infection rates. A negative jerk indicates a declining acceleration possibly including a shift from positive to negative acceleration, and if associated with a policy shift may indicate policy success. The 7-day persistence rate is the number of cases per 100,000 today that are statistically attributable to a case 7 days ago. A positive 7-day persistence rate can signify the presence of linked super-spreader events, the emergence of a new variant, or continued policy ineffectiveness. A negative 7-day persistence rate indicates that a high number of cases last week is associated with a lower number of cases this week, suggesting that the high number of cases was an aberration rather than an indication of a persistent issue in the metropolis. A negative 7-day persistence rate is indicative of a slowing pandemic, whether via a natural progression or through public health control measures.

Our use of a dynamic panel data on model is motivated by the observation that contagion models are typically population-based differential equations of the form ${dY}/{dt}=f(Y, X)$, where Y is a vector of population or sub-population characteristics of interest such as the number of exposed or infected individuals, X is a vector of mediating factors (often omitted), and f is a transition function. For empirical purposes we will use difference equations since the data come in discrete time periods, specifically days. For example, a basic component of susceptible-exposed-infected-recovered-deceased (SEIRD) models represents the number of new infections in a metropolitan area by

1. $I_{t}- I_{t-1} = {\beta S_{t-1}I_{t-1}}/{E_{t-1}}- {(\gamma}_{R}+\gamma_{D})I_{t-1}$

or a similar equation, where S, I and E are the sizes of the susceptible, infected, and exposed, populations, respectively; beta and the gammas are the unknown model parameters determining the infection rate, recovery rate, and death rate, respectively, and the *t* and *t-1* subscripts denote the time period. Calibration of contagion models requires estimates of the true parameter values.

The availability of county-level data suggests rewriting equation (1) in panel regression form as

(2) $I_{it} = \alpha_{i}+{(1+ \beta S_{it-1}}/{E_{it-1}}- {(\gamma}_{R}+\gamma_{D}))I_{it-1}+\varepsilon_{it}$.

The additional subscript *i* refers to the county, so that $I_{it}$ represents the number of infected people in county *i* at time *t*. Consistently with panel data specifications, we have added a state-specific ‘fixed effects’ $\alpha_{\cdot i}$ and error terms $\varepsilon_{\cdot it}$ to each of the equations. The primary advantage of the panel specification is that it captures information about the epidemiological curve from each county in the metropolitan area, whereas statistical modeling at the aggregate metropolitan area loses this information [26,27].

Conventionally, the epidemiologist calibrates these models through contact tracing including serological testing especially when there is a significant asymptomatic population. This approach is very accurate, but usually time consuming and expensive, even with a small sample size. For surveillance purposes, [7,26] propose a statistical approach based on publicly available infection data that essentially models the epidemiological curve for the number of reported COVID-19 cases in specified geographic locations.

We adapt the [7,26] statistical model to be

(3) $P_{it} = \alpha_{i}+\beta_{1}P_{it-1}{+ \beta}_{2}P_{it-2}+\sum_{w=1}^{6} \beta_{3w}I_{w}P_{it-7} +\varepsilon_{it}$ ,

where $P_{it}$ is the number of positively confirmed cases in county *i* at time *t* as reported by health officials, the subscript *w* denotes the week, and $I_{w}$ is an indicator variable designated whether the observation is in week *w* ($I_{w}=1)$ or not ($I_{w}=0)$. We group observations by week because reporting can vary across the day of the week. We include the seven-day lagged value $P_{it-7}$ because i) it approximately represents the median length of time for COVID-19 incubation plus a day or two for the patient to come in for testing and results to be reported, and ii) it works well empirically across the globe including in metropolitan U.S. [7,26-28,30,31,35]. The term $\beta_{3w}$ is the non-normalized 7-day persistence rate, which varies by week as indicated by the subscript w and is measured in cases per day [this week] per cases per day [7 days prior], and hence is unitless analogously to percentages. We multiply by the average number of cases per day the previous week and then normalize by population to obtain the (normalized) 7-day persistence rate, which is measured in units of new cases per day per 100,000 persons. There are four big issues with applying standard regression analysis to equation (3). First, the inclusion of lagged values of the dependent variable on the right side of the equation introduces autocorrelation in the error term, as well as possibly other inconsistencies with least-squares statistical assumptions. Hansen’s generalized method of moments (GMM) provides an alternative approach that essentially finds those parameter values which most closely fit the underlying statistical assumptions, as well as testing the statistical validity of that fit [36]. Second, the sparse parameterization means that variables representing mediating factors such as differences in local compliance with mask mandates (for which data are largely absent) as well as many others are omitted from the regression model. This means that the included variables could be picking up effects from the omitted variables, creating bias in the estimated parameters. The standard correction for this is an instrumental variables approach. Third, the relationships between reported cases over time even within a single county may change over time as the county imposes or removes public health measures, as public compliance changes, or as new variants emerge, among other influences. To address this issue, we focus on the most recent six weeks of data and allow for the seven-day lag parameter $\beta_{3w}$ to change weekly. Fourth, with a six-week time frame, we are possibly in the sphere of having a large number of cross-sectional observations and a small number of observations across time. Standard panel data methods have favorable properties when the time period is long (they behave well asymptotically as t→∞) but are inappropriate for samples with ‘small t’. The dynamic panel data corrects these issues using GMM with instrumental variables and corrections for autocorrelation. Statistical derivations of dynamic panel data models and their properties are available in many textbooks such as [34] and other teaching resources such as [37]; [38] provides an intuitive discussion of the advantages and use of dynamic panel data models.

A significant drawback to DPD methods is that they are computationally complex and become very time and resource intensive as the number of observations grows.

We collected data usafacts.org for the most recent eight weeks of 2021, i.e., the week ending on Aug. 1^st^, 2021 through the week ending on Sep. 19^th^, 2021. Using U.S. Census Bureau definitions of metropolitan area, we determine the various counties comprising the 25 largest U.S. metropolitan areas and collected data on the total (cumulative) number of COVID-19 cases in each country for each day county. We pre-processed the data only by creating a variable containing the number of new cases per county per day to be the change in the cumulative case count from the prior day to that day (first difference). We used data from the first two weeks, i.e., the weeks ended Aug. 1^st^, 2021 and Aug. 8^th^, 2021 to create lagged values used in the analysis, resulting in a sample period of six weeks from the week ended Aug. 15^th^, 2021 to the week ended Sep. 19^th^, 2021 for which we had a complete data set. We analyzed the data using STATA/MP 17.0; the dynamic panel data modeling is accomplished using STATA’s “xtabond” procedure. We report the weekly average speed, acceleration, jerk, and 7-day persistence effect for the most recent six weeks of 2021. Results for prior weeks of 2021 are available from the authors upon request.
